# harmonize-wq: Standardize, clean and wrangle Water Quality Portal data into more analytic-ready formats

**DOI:** 10.21105/joss.07305

**Published:** 2024-10-22

**Authors:** Justin Bousquin, Cristina A. Mullin

**Affiliations:** 1 U.S. Environmental Protection Agency, Gulf Ecosystem Measurement and Modeling Division, Gulf Breeze, FL 32561; 2 U.S. Environmental Protection Agency, Watershed Restoration, Assessment and Protection Division, Washington, D.C. 20460

## Summary

The U.S. EPA’s Water Quality Exchange (WQX) allows state environmental agencies, the EPA, other federal agencies, universities, private citizens, and other organizations to provide water quality, biological, and physical data (Read & Winslow, 2017). The Water Quality Portal (WQP) is a data warehouse that facilitates access to data stored in large water quality databases, including WQX, in a common format. WQP has become an essential resource with tools to facilitate both data publishing (WQX Web API, 2018; WQX Web User Guide, 2020) and data retrieval (De Cicco et al., 2022; Hodson & Horsburgh, 2023). However, given the variety of data originators and methods, using the data in analysis often requires cleaning to ensure it meets required quality standards and wrangling to get it in a more analytic-ready format. Although there are many examples where this data cleaning or wrangling has been performed (Bousquin, 2021; Evans & Malcom, 2021; Manning & Kominoski, 2020; Ross & Pavelsky, 2019; Shen & Domisch, 2020), standardized tools to perform this task will make it less time-intensive, more standardized, and more reproducible. More standardized data cleansing and wrangling allows easier integration of outputs into other tools in the water quality data pipeline, e.g., for integration into hydrologic analysis (Chegini et al., 2021), dashboards for visualization (Beck & Best, 2021) or decision support tools (Booth & Murphy, 2011).

## Statement of need

Due to the diversity of data originators metadata quality varies and can pose significant challenges preventing WQP from being used as an analysis-ready data set (Shaughnessy & Brantley, 2019; Sprague & Argue, 2017). Recognizing the definition of ‘analysis-ready’ varies depending on the analysis, our goal with harmonize-wq is to provide a robust, flexible, water quality specific framework that will help the data analyst identify differences in data units, sampling or analytic methods, and resolve data errors using transparent assumptions. Domain experts must decide what data meets their quality standards for data comparability and any thresholds for acceptance or rejection.

## Current Functionality

WQP is intended to be flexible in how data providers structure their data, what data they provide, and what metadata is associated with the data. The harmonize-wq package does not identify results for rejection, but it does flag those that were altered in a QA column. The package uses the metadata available to clean characteristic data into usable, comparable measures. Four data characteristics are the focus for cleaning the data:

- Measure – If missing (NAN) or not the correct data type, e.g., non-numeric and non-categorical, it cannot be used in analysis.
- Sample Fraction – A measure for a given WQP characteristic, e.g., Phosphorous, may have differences in the analyzed samples, e.g., filtered, dissolved, organic, inorganic, etc. Where these may make measures incomparable to one another results are split into sample fraction specific columns.
- Speciation/Basis/Standards - A measure for a given WQP characteristic, e.g., Nitrogen, may have differences in the molecular basis measured, e.g., ‘as NO3’ vs. ‘as N’. Likewise, some measures will differ depending on sample conditions, such as temperature and pressure. Since these differences will alter the comparability of results they are moved to the appropriate column for consideration in conversions and analyst decisions.
- Units - Units of measure are converted using Pint (Grecco & Chéron, 2021). To facilitate this, harmonize-wq defines new units, e.g., ‘NTU’ for turbidity, and updates WQP units for recognition by Pint, e.g., ‘deg C’ for water temperature is updated to ‘degC.’ Where units are missing (NAN) or unrecognized, an attempt is made to assume standard or user-specified units and a flag is added to the QA column. Pint contexts are used to change dimensionality of units, e.g., from mg/l (mass/volume) to g/kg of water (dimensionless), before final conversion. Some additional custom conversions were added, e.g., dissolved oxygen percent saturation to concentration in mg/l. When a unit is falsely recognized, e.g., ‘deg c’ recognized as degree * speed of light, it will typically result in a dimensionality error during conversion. The default is for conversion issues to error, but the user has the option to suppress that error, replacing the results with the un-converted units or as NAN.

In addition to cleaning characteristic results, the package also harmonizes metadata defining the observation. These metadata include site location – where geopandas (Kelsey Jordahl & Wasser, 2021) transforms points to a consistent datum, and time of observation – where dataRetrieval (Hodson & Horsburgh, 2023) interprets timezone.

Data wrangling involves reducing the complexity of the data to make it more accessible and reshaping the data for use in analysis. The WQP data format is complex, with each row corresponding to a specific result for a specific characteristic and many columns for metadata specific to that result. The harmonize-wq package reshapes the table to loosely adhere to tidy principles (Wickham, 2014), where each variable forms a column (i.e., one characteristic per column) and each observation forms a row (i.e., one row per site and time stamp). Given the number of result specific WQP metadata columns, to avoid conflicts during reshaping the package has functions to differentiate these based on the original characteristic, e.g., ‘QA’ becoming ‘QA_Nitrogen’. Once the data has been cleansed and result specific columns differentiated many of the original columns can be reduced. The package also has resources for entity resolution, both for deduplication when one source has duplicate results during reshaping (e.g., quality control or calibration sample) and when the same result is reported by different sources after the table is reshaped.

## References

[R1] Beck S, M. W., & Best BD (2021). tbeptools: An R package for synthesizing estuarine data for environmental research. Journal of Open Source Software, 6(65), 3485. 10.21105/joss.03485

[R2] Booth E, N. L., & Murphy L (2011). A Web-Based Decision Support System for Assessing Regional Water-Quality Conditions and Management Actions. Journal of the American Water Resources Association, 47 (5), 1136–1150. 10.1111/j.1752-1688.2011.00573.x 22457585 PMC3307623

[R3] Bousquin J. (2021). Discrete Global Grid Systems as scalable geospatial frameworks for characterizing coastal environments. Environmental Modelling & Software, 146, 105210. 10.1016/j.envsoft.2021.105210 PMC8958999 35355513

[R4] Chegini T, Li H-Y, & Leung LR (2021). HyRiver: Hydroclimate Data Retriever. Journal of Open Source Software, 6(66), 1–3. 10.21105/joss.03175

[R5] De Cicco LA, Lorenz D, Hirsch RM, Watkins W, & Johnson M (2022). dataRetrieval: R packages for discovering and retrieving water data available from U.S. federal hydrologic web services (Version 2.7.12) [Computer software]. U.S. Geological Survey; U.S. Geological Survey. 10.5066/P9X4L3GE

[R6] Evans K, M. J., & Malcom JW (2021). Linking mountaintop removal mining to water quality for imperiled species using satellite data. PloS One, 16(11), e0239691. 10.1371/journal.pone.0239691 34735447 PMC8568141

[R7] Grecco H, & Chéron J (2021). Pint: Operate and manipulate physical quantities in Python (Version 1.9). https://github.com/hgrecco/pint

[R8] Hodson H, T. O., & Horsburgh JS (2023). dataretrieval (Python): a Python package for discovering and retrieving water data available from U.S. federal hydrologic web services (Version 1.0.2). U.S. Geological Survey; U.S. Geological Survey. 10.5066/P94I5TX3

[R9] Kelsey Jordahl MF, Van den Bossche Joris, & Wasser L (2021). geopandas/geopandas: v0.10.2 (Version v0.10.2). Zenodo. 10.5281/zenodo.5573592

[R10] Manning R, D. W., & Kominoski JS (2020). Transport of N and P in US streams and rivers differs with land use and between dissolved and particulate forms. Ecological Applications, 30, p.e02130. 10.1002/eap.2130 32227394 PMC7507146

[R11] Read C, E. K., & Winslow LA (2017). Water quality data for national-scale aquatic research: The Water Quality Portal. Water Resources Research, 53, 1735–1745. 10.1002/2016WR019993

[R12] Ross T, M. R., & Pavelsky TM (2019). AquaSat: A data set to enable remote sensing of water quality for inland waters. Water Resources Research, 55, 10012–10025. 10.1029/2019WR024883

[R13] Shaughnessy W, A. R., & Brantley SL (2019). Three Principles to Use in Streamlining Water Quality Research through Data Uniformity. Environmental Science & Technology, 53, 13549–13550. 10.1021/acs.est.9b06406 31742393

[R14] Shen A, L. Q., & Domisch S (2020). Estimating nitrogen and phosphorus concentrations in streams and rivers, within a machine learning framework. Scientific Data, 7, 161. 10.1038/s41597-020-0478-7 32467642 PMC7256043

[R15] Sprague O, L. A., & Argue DM (2017). Challenges with secondary use of multi-source water-quality data in the United States. Water Research, 110, 252–261. 10.1016/j.watres.2016.12.024 28027524

[R16] Wickham H. (2014). Tidy data. The Journal of Statistical Software, 59, 252–261. 10.18637/jss.v059.i10

[R17] WQX Web API. (2018). [Computer software]. U.S. Environmental Protection Agency, Office of Water; U.S. Environmental Protection Agency. https://www.epa.gov/sites/default/files/2018-09/documents/wqx_web_application_programming_interface_api.pdf

[R18] WQX web user guide (Version 3.0). (2020). [Computer software]. U.S. Environmental Protection Agency, Office of Water; U.S. Environmental Protection Agency. https://www.epa.gov/sites/default/files/2020-03/documents/wqx_web_user_guide_v3.0.pdf

